# Characterization of the complete chloroplast genome of summer snowflake (*Leucojum aestivum*, Amaryllidaceae)

**DOI:** 10.1080/23802359.2018.1501309

**Published:** 2018-09-10

**Authors:** Meng-Di Li, Xue Wu, Jun-Jie Wu, Xuan Zhou, Rui-Hong Wang, Zhe-Chen Qi

**Affiliations:** a College of Life Sciences, Zhejiang Sci-Tech University, Hangzhou, China;; b Zhejiang Province Key Laboratory of Plant Secondary Metabolism and Regulation, Hangzhou, China

**Keywords:** Chloroplast genome, *Leucojum aestivum*, medicinal plant, phylogenomics

## Abstract

*Leucojum aestivum* (Amaryllidaceae) is an important medicinal plant native to Europe, North Africa, and Central Asia. Its wild resources are Endangered because of excavation. In the present study, the chloroplast genome of *L. aestivum* was sequenced. The plastome length is 157,241 bp. A total of 132 genes were identified, consisting of 86 protein-coding genes, eight rRNA genes, and 38 tRNA genes. Thirty-four species from Asparagales were used for phylogenomic analysis.

*Leucojum aestivum* L. (Amaryllidaceae), known as summer snowflake, is a perennial bulbous plant naturally distributed in the Europe, North Africa, and Central Asia. It is widely cultivated as an ornamental plant but more importantly as a medicinal plant for galanthamine, an acetylcholinesterase inhibitor for the treatment of Alzheimer’s disease (Heinrich and Lee Teoh 2004; Georgiev et al. 2012). *L. aestivum* is included in the IUCN Red List of Endangered species because of its depletion of wild populations for commercial demand. Recent studies show that the galanthamine content varies widely in different geographical populations and may related to its genotypes (Georgieva et al. 2007; Berkov et al. 2013). In this study, we assembled the complete plastome of *L. aestivum*. It is the first complete plastome reported in this genus. And it will provide potential genetic resources for further population genetics and evolutionary studies for this valuable medicinal plant and its relatives.

Fresh leaf samples were collected from Hangzhou Flower Nursery, Hangzhou, Zhejiang province, China (Voucher No. L20180318, deposited at Zhejiang Sci-Tech University). Total DNA was extracted using a modified CTBA method assisted with DNA Plantzol (Invitrogen, Carlsbad, CA). The plastome sequences were generated by Illumina HiSeq 2500 platform (Illumina Inc., San Diego, CA). Then CLC Genomics Workbench (CLC Bio, Aarhus, Denmark) was used for trimming, de novo assembly, and mapping to *Agapanthus coddii* (NC035971) as a reference. BLAST, GeSeq (Tillich et al. 2017), tRNAscan-SE v1.3.1 (Schattner et al. 2005), and GENEIOUS v 11.0.5 (Biomatters Ltd, Auckland, New Zealand) were used to align, assemble, and annotate the plastome.

The full length of chloroplast genome *L. aestivum* (GenBank accession No. GRP6609808) is 157,241 bp (with 37.9% GC content). It consist of a large single copy region (LSC, 85,657 bp, 36% GC content), a small single copy region (SSC, 18,180 bp, 32.1% GC content), and two inverted repeat regions (IR, 26,702, 42.9% GC content). There were 132 genes in *L. aestivum* cp genome, including 86 protein-coding genes, eight rRNA genes, and 38 tRNA genes. Among them, three protein-coding genes (*ndhB, rpl23, ycf2*), all four rRNA genes (*rrn5, rrn4.5, rrn23, rrn16*), and eight tRNA genes (*trnN-GUU, trnR-ACG, trnA-UGC, trnI-GAU, trnV-GAC, trnL-CAA, trnH-GUG, trnI-CAU*) have two copies. Nine of these protein-coding genes (*rps16, atpF, rpoC1, ndhB, petB, petD, rpl16, rpl2, ndhA*) have one introns and three (*ycf3*, *clpP*, *rps12*) of them have two.

To further test the phylogentic placement of *L. aestivum* in Asparagales, 34 completed chloroplast genomes (consist of *Agapanthus codii,* four *Allium* species, and 29 species from Asparagaceae) were selected for phylogenomic analysis. The plastome alignment was conducted using MAFFT v7.3 (Kazutaka and Standley 2013). The maximum likelihood (ML) inference was performed using GTR + I model with 1000 bootstrap replicates in RAxML v.8.2.8 (Stamatakis 2014). The result revealed that *L. aestivum* was clustered with a clade of *Allium* with 85% bootstrap values. And the whole Amaryllidaceae was clustered with Asparagaceae with 100% bootstrap values (Figure 1).

**Figure 1.**
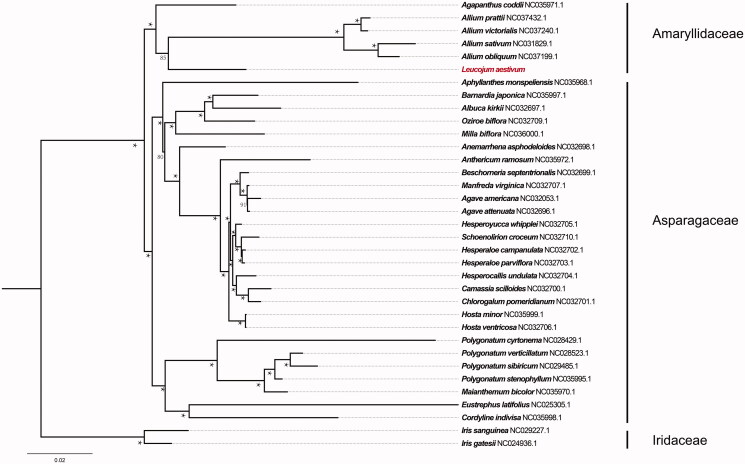
RAxML phylogeny of Asparagales based on 35 complete cp genomes (Accession numbers of were listed behind their names, and labels showed beside branches represent of bootstrap values, and ‘*’ indicates a 100% bootstrap value).
